# Preoperative treatment with dopamine agonist therapy influences surgical outcome in prolactinoma: a retrospective single-center on 159 patients

**DOI:** 10.1007/s00701-024-06198-5

**Published:** 2024-08-01

**Authors:** Alice Ryba, Diego Gonzalez Lopez, Roman Rotermund, Jörg Flitsch

**Affiliations:** 1 Department of Neurosurgery, Medical Center Hamburg-Eppendorf, Martinistraße 52, 20253 Hamburg, Germany; 2 Department of Neurosurgery, Diako Krankenhaus, Flensburg, Germany

**Keywords:** Prolactinoma, Transsphenoidal surgery, Dopamine agonist, Microadenoma, Macroadenoma

## Abstract

**Introduction:**

Prolactinoma account to the most common pituitary adenomas and current therapy regime constitutes of dopamine agonist therapy (DA) and surgery in selected cases [17]. Due to tumor fibrosis induced by previous DA therapy, surgical removal can be challenging though. Therefore, this study investigates how preoperative DA usage influences perioperative treatment and surgical outcome in prolactinoma and aims to ascertain whether a specific subgroup of prolactinoma patients could derive greater benefit from exclusive surgical intervention.

**Methods:**

We retrospectively analyzed n = 159 surgically treated and histologically confirmed prolactinomas in the sella region from 2013–2022 in our institution. Clinical, radiological and surgical features were analyzed. Univariate and multivariate analyses were performed.

**Results:**

Out of total of 159 prolactinoma patients, 83.6% received previous treatment with DA followed by surgery, while only 16.4% received exclusive surgery. Both groups presented similar initial tumor volumes (1.9cm^3^ vs. 1.5cm^3^, p = 0.59) and equal preoperative prolactin levels (PRL) (199.7 µg/l vs. 191.0 µg/l, p = 0.44). Surgical procedures took significantly longer when patients received prior DA treatment (79 min. vs. 70 min., p = 0.0479). Six months after surgery, pretreated patients revealed significantly higher PRL compared to non-treated (107 g/l vs. 8.64 µg/, p = 0.0009). Additionally, untreated microprolactinoma presented a remission of 100%, whereas pretreated exhibited a remission rate of 88.75%.

**Conclusion:**

The current study demonstrates that prior DA treatment is associated with significantly longer surgeries, higher recurrence rates and lower rates of normalization of PRL levels after surgery, particularly in microprolactinomas and support the latest recommendations of the Pituitary Society's Consensus Statement 2023, which favors the option of surgery alone as first-line therapy for microprolactinomas.

## Introduction

Prolactinoma are characterized by the excessive secretion of prolactin and account for approximately 30–45% of all pituitary adenomas, they represent the most common type of functional pituitary adenomas [3, 14]. These adenomas occur more often in female aged 20–50 years with a ratio of 10:1 [6]. Female patients typically experience a more benign clinical course, while their male counterparts often contend with more aggressive prolactinoma and the potential resistance to dopamine agonists (DA) [1, 4].

Since dopamine agonists, such as cabergoline and bromocriptine, have emerged as primary pharmacological agents in the management of prolactinomas, surgical removal of prolactinoma was then reserved for failed medical therapy only [6]. Recently, new guidelines have extended surgical indications in firstline treatment for selected cases of microprolactinoma and well-circumscribed macroprolactinoma with low Knosp grading (0–1), however medical treatment should especially be given to patients with low chance of surgical remission (Knosp > 2). DAs efficacy in reducing prolactin secretion resulting in normalized hormone levels in about 90% and inducing tumor shrinkage in 70–90% has positioned them as the cornerstone of medical treatment for prolactinoma patients [22].

Although DA alone effectively regulate tumor growth and hormone secretion in the majority of cases, a subset of patients may experience tumor progression despite DA therapy resulting in visual deterioration or may suffer from medical side-effects [18]. Especially in such scenarios, surgical intervention becomes imperative [2, 20]. Transsphenoidal surgery aims for complete tumor resection; in cases that are difficult to access, however, the remaining surgical goal is optic nerve decompression and tumor debulking.

Structural changes in tumor tissue, such as tumor fibrosis, induced by DA therapy can potentially complicate surgical excision [2, 10]. This raises the question whether dopamine agonist therapy preoperatively influences the surgical outcome and whether there might be a subgroup of prolactinoma patients who could benefit from surgical therapy as a first-line treatment.

## Methods

This retrospective study included a total of n = 159 patients who underwent transsphenoidal surgery for prolactinoma at our institution between 2013 and 2022. A comprehensive range of clinical parameters, radiological, surgical and histopathological parameters as well as its outcome were analyzed in regard of their preoperative treatment with dopamine agonists. Inclusion criteria for this study were histopathologically confirmed prolactinoma, together with patients who underwent transsphenoidal surgery and were aged > 18 years. The study was approved by the local ethics committee (2023–300417-WF) and performed in accordance with the ethical standard of the 1964th Helsinki declaration. Informed consent was obtained from all patients.

### Patient characteristics

Patient demographics and clinical characteristics were retrospectively extracted from hospital records. General characteristics included age, gender and preoperative clinical deficits such as visual impairment and hypopituitarism. Visual impairment is defined as occurrence of visual field deficits and/or blurry vision. Preoperative prolactin levels (PRL) in blood were measured one day prior to surgery in µg/l. Preoperative exposure to dopamine agonist (DA), its weekly dosage in mg, and the total duration of intake up to the time of surgery were collected. Furthermore, the cause of the change in treatment regimen from initial drug therapy to surgical resection was recorded. Postoperatively, major and minor surgical complications were documented and the necessity for adjuvant therapy following surgery was assessed. In the follow-up, PRL was examined 6 months after surgery and every following year afterwards. Discontinuation and/or reduction of DA therapy postoperatively was assessed. Recurrence was either measured on tumor progression/recurrence on MRI or increasing PRL in blood.

### Radiographic

All available magnet-resonance imaging (MRI) scans of the patients were analyzed for tumor volume and calculated with the estimation formula v = (l*w*h)/2. Additionally, Knosp score was assigned as an indicator of radiographic invasion.

### Surgical analysis

The surgical approach chosen was an endonasal microscopic or since 2019, 3D-videomicroscopic transsphenoidal technique and performed by expert pituitary neurosurgeons. Surgical duration was quantified in minutes, commencing from the first incision to the final closure.

### Histopathological Analysis

Patient inclusion was contingent on histopathological confirmation of prolactinoma diagnosis. Additionally, Ki-67 status and the presence of dural invasion were examined according to WHO standards.

### Statistical analysis

Both univariate and multivariate analyses were conducted using GraphPad Prism 9 software. Continuous scale data were assessed using an unpaired two-tailed or in selected cases one-tailed Student’s t-test, while two-group comparison employed the two-sided Fisher’s exact test. Progression-free survival (PFS) was evaluated using Kaplan–Meier method and univariate PFS analysis was performed using log-rank test. PFS was calculated from the time of first intervention. P value was considered statistically significant < 0.05.

## Results

### Patient and tumor characteristics

A total of 159 patients with a histologically confirmed prolactinoma in the sella region were identified between 2013 and 2022. Their characteristics are summarized in Table 1.

**Table 1:** Patient characteristic of the whole cohort of prolactinoma

Parameters		All (n = 159)
Clinical Data		
	Mean Age, yrs (SD)	37 (± 14.41)
	Sex ratio (M/F)	1:1.48
	Male, n (%)	64 (40.2)
	Female, n (%)	95 (59.8)
	Mean initial Prolactin Level (PRL), µg/l (SD)	197.9 (± 823.1)
	Preoperative visual impairment, n (%)	12 (7.5)
	Mean duration of surgery, min (SD)	77 (± 31.17)
Dopamine-agonist therapy		
	Preoperative DA-Intolerance, n (%)	48 (36.1)
	Preoperative DA-Resistance, n (%)	85 (63.9)
	Mean time until surgery, yrs (SD)	5.16 (± 6.46)
	Preoperative DA dose [mg/week]	2.81 (± 5.99)
Radiographic		
	Mean volume, cm^3^ (SD)	3.14 (± 6.85)
	Microprolactinoma, n (%)	92 (57.8)
	Macroprolactinoma, n (%)	64 (42.2)
	Knosp grading	
	0	20 (12.6)
	1	25 (15.7)
	2	8 (5)
	3	0 (0)
	4	6 (3.8)
	NA	100 (62.9)
Complications		
- Major complications		
	Internal carotid artery injury, n (%)	1 (0.6)
	ICU stay, n (%)	1 (0.6)
	CSF rhinorrhea, n (%)	3 (1.9)
	Death, n (%)	0 (0)
- Minor complications		
	Transient symptomatic SIADH, n (%)	10 (6.2)
	Transient DI, n (%)	7 (4.4)
Outcome		
	Mean follow-up after surgery, yrs (SD)	1.59 (± 2.06)
	Recurrence on MRI, n (%)	13 (8.2)
	Postoperative discontinuation of DA, n (%)	127 (79.8)
	Mean PRL after 6 Months, µg/l (SD)	88.86 (± 445.22)
	Normal PRL in FU, n (%)	67 (42.1)
	Adjuvant therapy, n (%)	9 (5.6)
	Radiation/Radio-surgery, n (%)	6 (3.7)
	Surgery, n (%)	2 (1.3)
	Chemotherapy, n (%)	1 (0.6)
	Mean PFS, yrs (SD)	2.52 (± 2.5)

The mean age at surgery reached 37 ± 14.41 years. The mean preoperatively measured PRL was at 197.9 µl/l. Female to male ratio was 1.48:1 (female 59.8%, male 40.2%). In the whole cohort, male presented a higher mean tumor volume compared to female (0.77 ± 1.9cm^3^ vs. 2.72 ± 4.6cm^3^, p = 0.0004). Furthermore, the mean Ki-67 status did not differ within gender (2.97% vs. 3.01%, p = 0.9203). Preoperative visual impairment was present in 7.5% of all patients (n = 12).

A total of 83.6% (n = 133) of patients received a therapy of DA preoperatively, mainly with cabergoline (CAB), while 16.3% of all patients underwent surgery without prior DA therapy. The majority of pretreated patients displayed DA resistance (63.9%), whereas only 36.1% underwent surgery due to DA intolerance accompanied by side effects. Mean weekly cumulative dosage of cabergoline was at 2.81 ± 5.99 mg and there was no significant difference in the weekly cumulative DA dosage between microadenoma and macroadenoma (2.5 mg vs. 3.1 mg, p = 0.5821). Additionally, the time elapsed from diagnosis until surgery was notably longer for the pre-treated group than for the non-treated group (6.0 years vs. 1.03 years, p = 0.0006). Differences in between the two groups of pretreated and non-treated prolactinoma are summarized in Table 2. Additionally, a further subanalysis of micro- and macroprolactinomas, including their differences with prior DA treatment before surgery, is summarized in Table 3.

**Table 2:** Differences of pretreated and non-treated prolactinoma in regard to perioperative parameters. Percentages are calculated within each group. P-values marked with (*) are performed as a one-sided t-test

Parameters		Non-treated (n = 26)	Pretreated (n = 133)	p-value
Clinical Data				
	Mean Age, yrs (SD)	39.69 (± 15.17)	36.5 (± 14.27)	0.3038
	Sex ratio (M/F)	1.36:1	1:1.66	0.049
	Male, n (%)	15 (57.7)	50 (37.6)	
	Female, n (%)	11 (42.3)	83 (62.4)	
	Mean initial Prolactin Level (PRL), µg/l (SD)	199.7 (± 90.73)	191.0 (± 64.24)	0.4452 *
	Preoperative visual impairment, n (%)	4 (11.1)	8 (6.2)	0.1103
	Mean Ki67, % (SD)	3.16 (± 1.6)	2.96 (± 2.4)	0.6926
Surgical				
	Mean duration of surgery, min (SD)	70.04 (± 24.21)	78.55 (± 32.26)	**0.0479** *
	Mean time until surgery, yrs (SD)	1.03 (± 1.41)	6.0 (± 6.75)	**0.0006**
Radiographic				
	Mean volume, cm^3^ (SD)	1.89 (+ 3.8)	1.49 (± 3.3)	0.5947
	Microprolactinoma, n (%)	14 (53.8)	80 (60.2)	0.6634
	Macroprolactinoma, n (%)	12 (46.2)	53 (39.8)	
	Knosp grading			0.6109
	0	2 (7.7)	18 (13.5)	
	1	3 (11.5)	22 (16.5)	
	2	2 (7.7)	6 (4.5)	
	3	0 (0)	0 (0)	
	4	0 (0)	6 (4.5)	
	NA	19 (73.1)	81 (61)	
Complications		5 (19.2)	17 (12.8)	0.3282
- Major complications				
	Internal carotid artery injury, n (%)	0 (0)	1 (0.8)	
	ICU stay, n (%)	0 (0)	1 (0.8)	
	CSF rhinorrhea, n (%)	1 (3.8)	2 (1.5)	
	Death, n (%)	0 (0)	0 (0)	
- Minor complications				
	Transient symptomatic SIADH, n (%)	2 (7.7)	8 (6.0)	
	Transient DI, n (%)	2 (7.7)	5 (3.8)	
Outcome				
	Mean follow-up after surgery, yrs (SD)	1.42 (± 1.5)	1.63 (± 2.1)	0.6627
	Recurrence on MRI, n (%)	3 (11.5)	22 (16.5)	0.7688
	Mean PFS, yrs (SD)	2.86 (± 0.0)	2.49 (± 2.65)	0.89
	Postoperative discontinuation of DA, n (%)	24 (92.3)	103 (77.4)	0.0787
	Mean PRL postoperatively			
	after Day 1, µg/l (SD)	11.40 (± 18.93)	19.07 (± 63.53)	0.1939
	after Day 3, µg/l (SD)	12.05 (± 21.93)	17.65 (± 54.59)	0.3077 *
	after 6 Months, µg/l (SD)	8.64 (± 4.98)	107.00 (± 491.9)	**0.0009** *
	Normal PRL in FU, n (%)	19 (73.1)	49 (36.8)	**0.0028**
	Adjuvant therapy, n (%)	0 (0)	9 (6.9)	0.18
	Radiation/Radio-surgery, n (%)	0 (0)	6 (4.6)	
	Surgery, n (%)	0 (0)	2 (1.5)	
	Chemo, n (%)	0 (0)	1 (0.6)	

^*^ one tailed t-test

**Table 3:** Subanalysis of micro- and macroprolactinoma and their differences after or no DA treatment priot to surgery in regard to perioperative parameters. Percentages are calculated within each group. P-values marked with (*) are performed as a one-sided t-test. Bolded p-values indicate significant differences

Parameters		Pretreated	Nontreated		Pretreated	Nontreated	
		Microprolactinoma	Microprolactinoma	p-value	Macroprolactinoma	Macroprolactinoma	p-value
Clinical Data		n = 80	n = 14		n = 53	n = 12	
	Mean Age, yrs (SD)	33.4 ± 9.9	40.4 ± 13.9	0.032	41.6 ± 18.1	39.2 ± 17.2	0.6652
	Sex ratio (M/F)	1:3.2	1:1	0.0556	1.4:1	2:1	0.7488
	Male, n (%)	19 (23.7)	7 (50)		31 (58.5)	8 (66.7)	
	Female, n (%)	61 (76.3)	7 (50)		22 (41.5)	4 (33.3)	
	Mean initial Prolactin Level (PRL), µg/l (SD)	70.4 ± 61.7	37.9 ± 46.5	0.0638	197.1 ± 313.6	206.5 ± 329.3	0.9267
Surgical							
	Mean duration of surgery, min (SD)	73.9 ± 21.9	61.1 ± 13.1	**0.0183** *	86.0 ± 42.6	80.1 ± 30.1	0.6727
Radiographic							
	Knosp grading, n (%)			> 0.9999			> 0.9999
	< 1	25 (31.3)	3 (21.4)		13 (24.5)	1 (8.3)	
	> 2	0 (0)	0 (0)		12 (22.6)	2 (16.7)	
	NA	55 (68.7)	11 (78.6)		28 (52.9)	9 (75)	
Complications		14 (17.5)	2 (14.3)	> 0.9999	3 (5.7)	3 (25)	0.0712
- Major complications							
	Internal carotid artery injury, n (%)	1 (1.3)	0 (0)		0 (0)	0 (0)	
	ICU stay, n (%)	1 (1.3)	0 (0)		0 (0)	0 (0)	
	CSF rhinorrhea, n (%)	0 (0)	1 (7.1)		2 (3.8)	0 (0)	
- Minor complications							
	Transient symptomatic SIADH, n (%)	8 (10)	1 (7.1)		0 (0)	1 (8.3)	
	Transient DI, n (%)	4 (5)	0 (0)		1 (1.9)	2 (16.7)	
Outcome							
	Mean follow-up after surgery, yrs (SD)	1.5 ± 2.1	1.6 ± 1.7	0.9164	1.8 ± 2.3	1.2 ± 2.4	0.4139
	Recurrence on MRI, n (%)	9 (11.3)	0 (0)	0.0005	13 (24.5)	3 (25)	0.2690
	PFS, Hazard Ratio (95%, CI)	3.4 (0.62—18.79)		0.2054	0.54 (0.12—2.51)		0.4346
	Postoperative DA, n (%)						
	Discontinued	72 (0.9)	14 (100)	0.6005	31 (58.5)	10 (83.4)	0.1883
	Reduced	5 (6.3)	0 (0)		12 (22.6)	0 (0)	
	Equal	0 (0)	0 (0)		3 (5.7)	0 (0)	
	Increased	0 (0)	0 (0)		0 (0)	1 (8.3)	
	NA	3 (3.8)	0 (0)		7 (13.2)	1 (8.3)	
	Mean PRL postoperatively, µg/l (SD)						
	after Day 1	5.8 ± 8.4	3.4 ± 4.1	0.3231	40.52 ± 98.4	20.4 ± 24.63	0.4800
	after Day 3	6.3 ± 9.8	4.7 ± 4.7	0.5671	34.8 ± 82.8	21.4 ± 30.9	0.6017
	after 6 Months	21.4 ± 24.1	6.4 ± 1.9	0.0575	200.3 ± 702.6	11.8 ± 6.4	0.4861
	Normal PRL in FU, n (%)	30 (37.5)	11 (78.6)	**0.0132**	19 (35.8)	8 (66.7)	0.0879
	Adjuvant therapy, n (%)	1 (1.3)	0 (0)	> 0.9999	8 (15.1)	0 (0)	0.3333
	Radiation/Radio-surgery, n (%)	0 (0)	0 (0)		6 (11.3)	0 (0)	
	Surgery, n (%)	1 (1.3)	0 (0)		1 (1.9)	0 (0)	
	Chemo, n (%)	0 (0)	0 (0)		1 (1.9)	0 (0)	

### Pretreated vs. Non-Treated

## Characteristics

At onset, pre-treated and non-treated prolactinoma shared the same baseline conditions. The pretreated cohort tended to represent slightly more female patients than male patients compared to the non-treated cohort. Both groups showed no significant age differences in mean age at time of admission to hospital (39.7 years vs. 36.5 years, p = 0.3038). However, within the microprolactinoma, the pretreated cohort presented a significantly lower mean age at time of admission (33.4 vs. 40.4 years, p = 0.032). Furthermore, the mean Ki-67, an indicator of cell proliferation, exhibited no marginal differences (2.96% vs. 3.16%, p = 0.6926). Of the 133 patients with prior DA treatment, 39 (29.3%) discontinued their medication before surgery and among these, more than half (27 out of 39, 69.2%) reported drug intolerance. Herein, the median duration of discontinuation prior to surgery was 6.4 months. The mean follow-up period after surgery was 1.63 years for the pretreated group and 1.42 years for the non-treated group. Initial prolactin levels (PRL) showed only minor variations in the two groups (199.7 µg/ml vs. 191.0 µg/ml, p = 0.4452). Preoperative visual impairment was observed in 6.2% of the pretreated group and 11.1% of the non-treated group. In terms of radiographic findings, the non-treated group presented an equal distribution of micro- and macroadenoma, whereas the pretreated group exhibited slightly, but not significantly more microadenoma (p = 0.6634). Additionally, the mean volume of lesions in the non-treated prolactinoma showed a slightly, but not significantly larger volume compared to pretreated prolactinoma (1.89cm^3^ vs. 1.49cm^3^, p = 0.5947).

### Outcome

**Figure 1:**
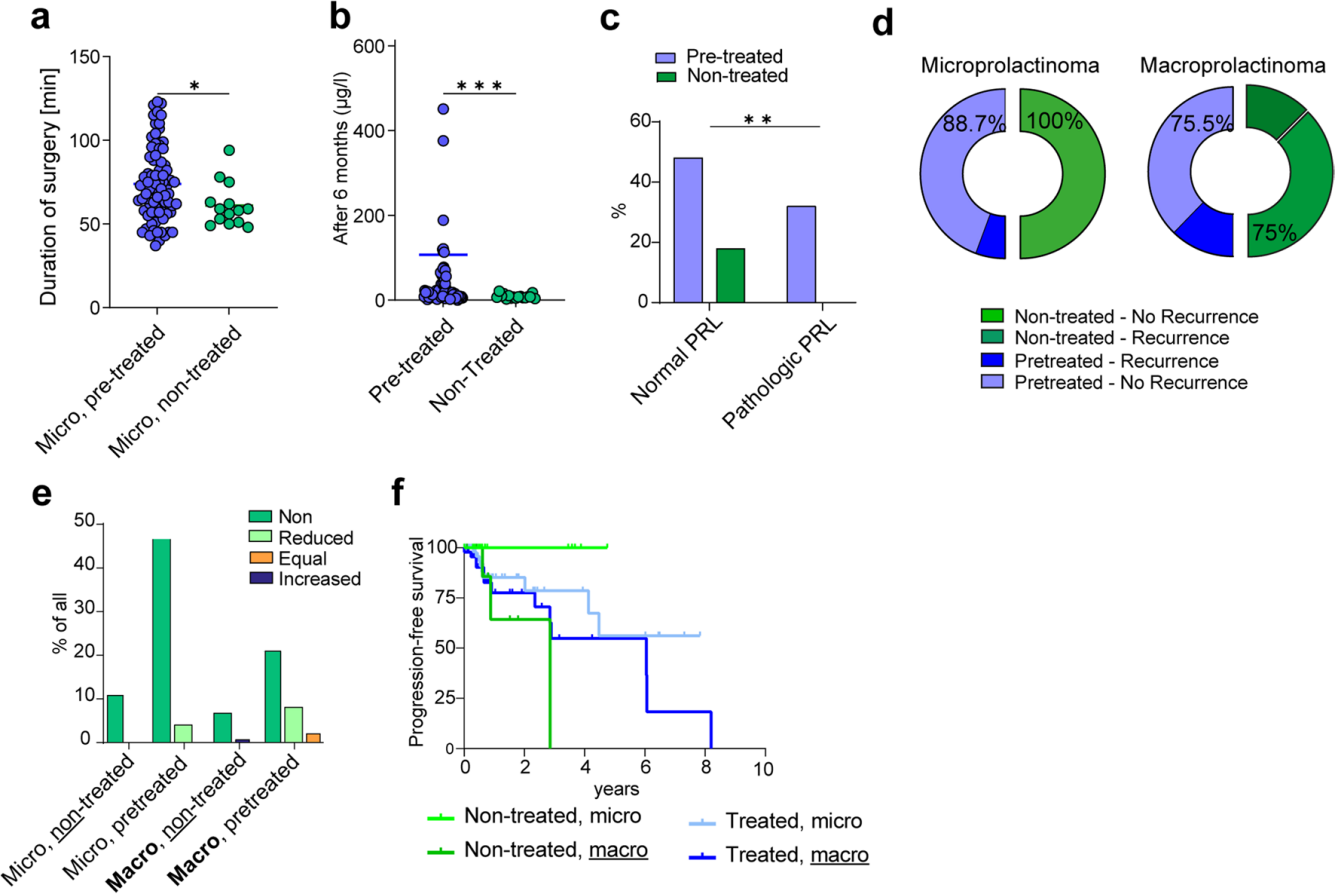
Outcome parameters after prior treatment with DA **a)** Microprolactinoma exhibit a significantly shorter duration of surgery when not exposed to DA preoperatively (61.1 vs. 73.9 min, p = 0.0183). **b)** After 6 months of follow-up, pretreated patients showed a greater proportion of pathologically elevated prolactin levels compared to non-treated patients (p = 0.0028). **c)** After 6 months of follow-up, prolactin levels (PRL) were significantly elevated in the pre-treated group compared to the non-treated cohort (73.1% vs. 36.8%, p = 0.0028). **d)** Radiographic recurrence was observed in 0% (n = 0) of the untreated macroprolactinoma, compared to 11.3% (n = 9) of pretreated microprolactinoma (p = 0.0005). In macroprolactinoma, recurrence on MRI occurred in 25% of patients in the untreated cohort and in 24.5% in the non-treated group (p = 0.2690). **e)** Bar chart of postoperative DA therapy in different groups: in 8.3% (n = 1) of all non-treated macroadenoma a postoperative DA therapy needed to be implemented. In 5.7% of all pretreated macroadenoma postoperative DA therapy did not change and 58.5% of these patients were able to discontinue their medication or reduce the dosage after surgery in 22.6% of the cases. **f)** Non-treated macroprolactinoma tended to show a slightly, but not significantly shorter PFS compared to pretreated macroprolactinoma (2.86 years vs. 6.05 years, p = 0.4346)

The mean duration of surgery was 77 min, however pretreated patients exhibited significantly longer surgeries (70 ± 24.2 min vs. 78.5 ± 32.2 min, p = 0.0479). Patients that discontinued their previous DA medication before surgery demonstrated no significant changes in the duration of surgery (71.7 vs. 81.6 min, p = 0.0501). However, in a further subanalysis focusing exclusively on micro- and macroprolactinomas, we found that microprolactinomas had a significantly shorter duration of surgery when not exposed to DA preoperatively (61.1 vs. 73.9 min, p = 0.0183) (Fig. 1a).

With regard to postoperative complications, major adverse events were infrequent and showed only subtle disparities between the two groups. Adverse events that occurred could be subdivided into major complications and minor complications. Major complications included internal carotid artery injury, which appeared in one patient (0.8%) in the pretreated group and 0% in the non-treated cohort, the necessity of an ICU stay (0.8% in the pretreated group, 0% in the non-treated group), CSF rhinorrhea (1.5% in the pretreated group and 3.8% in the non-treated group) as well as death (0% in both groups). Minor complications included transient symptomatic SIADH, which appeared in 6% in the pretreated group and in 7.7% in the non-treated group and transient diabetes insipidus (DI) accounted 3.8% in the pretreated group and 7.7% in the non-treated group.

In addition, the two different groups showed a similar decrease in blood PRL on the 1st and 3rd day after surgery. After six months, however, the pretreated group had a significantly higher mean PRL, whereas the non-treated group showed a significantly lower mean PRL (107.00 µg/l vs. 8.64 µg/l, p = 0.0009) (Fig. 1b). Furthermore, a significantly higher fraction of patients of the non-treated cohort presented a normalized PRL after 6 months of follow-up (73.1% vs. 36.8%, p = 0.0028) (Fig. 1c). Radiographic recurrence appeared significantly more often in pretreated microprolactinoma compared to untreated microprolactinoma (11.3% vs. 0%, p = 0.0005) (Fig. 1d). In contrary, in macroprolactinoma, the recurrence on MRI was similar for the pretreated cohort compared to non treated cohort (25% vs. 24.5%, p = 0.2690) (Fig. 2d). Additionally, in the non-treated microadenoma there was no need to initiate DA therapy postoperatively in a 100% of these cases, however in the pretreated microadenoma an already ongoing therapy had to be continued, but the dosage could be reduced in 6.8% and 93.2% were able to drop off their medication completely after surgery (Fig. 1e). In macroadenomas, however, the non-pretreated cohort showed one patient (8.3%), that needed to start DA therapy due to ongoing hyperprolactinemia. In the pretreated macroadenomas, therapy could be stopped in 58.5% of all cases postoperatively, whereas 22.6% had to continue therapy but were able to reduce dosage, and 5.7% continued DA therapy without dose modification (Fig. 1e).

Adjuvant therapies, including radiation, relapse surgery, and chemotherapy, were administered more frequently, but not significantly in the pretreated group (6.9%) than in the non-treated group (0%) (p = 0.18).

Untreated prolactinoma showed a recurrence in up 11.5% of cases, all of which were macroprolactinoma. In contrast, prolactinomas that received prior treatment exhibited recurrences in up to 16.5%. Among these cases, 40.9% were attributed to microprolactinomas, while 59.1% were linked to macroprolactinomas. Overall, there were no significant differences in the PFS between pretreated and non-treated prolactinomas (2.86 vs. 2.49, p = 0.89). However, in subgroup analyses focusing exclusively on macroprolactinoma, we found that non-treated macroprolactinoma tended to show a slightly, but not significantly shorter PFS compared to pretreated macroprolactinoma (2.86 years vs. 6.05 years, p = 0.4346) (Fig. 1f).

## Discussion

Prolactinoma are among the most common pituitary adenomas and first-line treatment is conventional treatment with dopamine agonist (DA) [6]. Only a subset of around 10% of all prolactinoma are considered DA-resistant and are characterized by a drug-induced tumor reduction of less than 50% and persistent hyperprolactinemia [18]. In case of DA resistance or in rare cases of DA intolerance, transsphenoidal surgery represents a valid alternative method.

Patients indicated for surgery in our series failed prior medical therapy due to medical side effects in 36.1% and inadequate medical response in 63.9% of the cases. A total of 16.3% of the patients underwent surgery without prior dopaminergic treatment. In a series of Primeau et al. drug intolerance appeared in 21% of all cases, whereas medical resistance was more common with 41% [17]. Other studies indicated a similar amount of around 10–20% of patients, who received exclusive surgery as first-line treatment without prior medical attempt [9, 17]. We discovered a more balanced sex ratio in our surgical cohort, which contrasts with the usual female predominance of this disease. According to our data, males had a significantly higher tumor volume compared to females (2.72 ± 4.6 cm^3^ vs. 0.77 ± 1.9 cm^3^, p = 0.0004), potentially influencing surgical decision making in male patients despite the natural female predominance in prolactinoma.

In our total cohort, we reported 79.8% of patients, who were able to stop their dopaminergic medication after surgery and 91.8% of patients did not depict any recurrence on MRI. In other studies, the remission rate varied from 40–70% and was widely varying with tumor size and its invasiveness [8, 13, 17, 24]. Interestingly, in our series, pretreated patients exhibited significantly higher postoperative prolactin levels after 6 months of follow-up compared to their untreated counterparts.

Several studies have already described that preoperative dopaminergic therapy leads to tumor fibrosis in prolactinoma and thus may complicate surgical outcome and increase the risk of perioperative complications [7, 12]. In the present study, we demonstrated that prolactinoma pretreated with DA resulted in a prolonged duration of surgery compared to those who had not received prior treatment. This suggests that changes induced by DA therapy might indeed impact the surgical process, potentially making tumor removal more challenging.

Notably, we were able to demonstrate a distinct benefit for untreated microprolactinoma of exclusive surgery. None of these patients displayed any recurrence on postoperative MRI. Furthermore, during the follow-up period after surgery, none of the cases required initiation of adjuvant DA therapy, demonstrating a successful surgical outcome for microprolactinoma. Previous studies were also able to show similar results for microprolactinoma solely, depicting a long-term remission rate of up to 70–100% [5, 21]. Additionally, perioperative complication rates were described low and make up only 0 to 5% [11, 19]. Since microprolactinoma clearly benefit from surgical removal, it remains debatable whether surgical first-line therapy may be a better option for patients to 1) allow the patient to recover from his/her benign tumor disease relatively quickly and with low surgical complication rates and 2) avoid possible side effects of lifelong, dopaminergic medication. Therefore, our findings are in accordance with the 2023rd Consensus statements of the Pituitary Society recommending surgery over medical treatment in patients with microprolactinoma or well-circumscribed macroprolactinoma with a Knosp-grade ≤ 1 [15].

In contrast, among patients with macroprolactinoma, the outcomes were less definitive. In our study, a significant percentage of patients of the untreated cohort experienced recurrence as detected by MRI or required initiation of DA therapy postoperatively. With regard to the literature, DA-resistance tends to occur more commonly in invasive macroprolactinoma [23]. Especially large and invasive tumors represent a challenge for an extensive surgical resection, though extent of resection remains as a major predictor for postoperative remission in prolactinoma. Previous studies showed a normalized PRL in only 10% of all surgically approached macroprolactinoma [23]. Pretrossians et al., showed that a surgical debulking accompanied by DA therapy, helped to reduce the DA dosage postoperatively to 50% and then by combining medical and surgical therapy decrease PRL significantly [16]. Especially in the case of macroprolactinomas, the frequent occurrence of drug therapy failure and inadequate cure rates with surgery, as well as significant side effects of adjuvant radiation, demonstrate the indispensable need for further research into new therapeutic approaches.

Additionally, the study's surgical follow-up is limited to 1.59 years. According to the latest recommendations from the 2023 Consensus Statement on prolactinoma treatment, MR imaging is recommended 3–6 months after surgery [15]. Subsequent imaging is only advised for partially resected DA-resistant prolactinomas or fully resected prolactinomas if there is an increase in PRL, which might explain the reduced surgical follow-up period of 1.59 years.

The study contributes valuable insights into the management of prolactinomas by examining the impact of DA pretreatment on surgical outcome. While DA remain the recommended initial therapy for prolactinomas, the study highlights potential complexities introduced by pretreatment of DA, particularly in the context of surgery. The findings underscore the need for personalized treatment strategies based on tumor characteristics and patient profiles.

## Conclusion

The present study is one of the largest, existing single-center studies investigating the effects of preoperative dopaminergic medication before surgery. In conclusion, the present study emphasizes that medical pretreatment with DA prior to prolactinoma surgery significantly prolongs overall surgery time, suggesting that surgery in pre-treated patients might be more challenging. Additionally, our study demonstrated that untreated prolactinomas showed favorable postoperative outcomes, including significant normalization of PRL levels, significantly lower PRL levels, and a significantly lower recurrence rates. The impact of pre-treatment on surgery and postoperative outcomes appears primarily attributable to microprolactinomas, with an additional notable statistical trend in macroprolactinomas.These outcomes contribute to a deeper understanding of how pretreatment with DA might impact the course and outcomes of prolactinoma cases and underscore the latest Consensus Statement of the Pituitary Society of surgical treatment for mircoprolactinoma and well-circumscribed macroprolactinoma with a Knosp grade from 0 to 1.

## Limitations

This study is a single-center study with retrospective data analysis. In addition, the cohort of non-treated patients is relatively smaller due to the common therapy regime using primarily conservative DA therapy, so this needs to be tested in a larger setting. Additionally, due to the retrospective characteristics of this study, the availability of radiographic data, including the Knosp grading, is limited. Moreover, we found a higher proportion of male patients in both cohorts, with this difference being even more pronounced in the untreated cohort, which is atypical given the usual female predominance of this disease.

## Data Availability

The data supporting the findings of this study are available from the corresponding author upon reasonable request.

## Abbreviations

DA: Dopamine Agonist
PRL: Prolactin level
CAB: Cabergolin
DI: Diabetes insipidus
SIADH: Syndrome Of Inappropriate Diuretic Hormone Secretion
MRI: Magnet Resonance Imaging
L: Length
W: Weight
H: Height
PFS: Progression-free Survival
ICU: Intensive Care Unit
CSF: Cerebrospinal fluid
